# Integrated Physiological, Transcriptomic and Metabolomic Analyses of the Response of Rice to Aniline Toxicity

**DOI:** 10.3390/ijms26020582

**Published:** 2025-01-11

**Authors:** Jingjing Wang, Ruixin Wang, Lei Liu, Wenrui Zhang, Zhonghuan Yin, Rui Guo, Dan Wang, Changhong Guo

**Affiliations:** Key Laboratory of Molecular Cytogenetics and Genetic Breeding of Heilongjiang Province, College of Life Science and Technology, Harbin Normal University, No. 1, Shida Road, Limin Economic and Technological Development Zone, Harbin 150025, China; swxwjj@126.com (J.W.); hsd_wangruixin@stu.hrbnu.edu.cn (R.W.); liulei1996@hrbnu.edu.cn (L.L.); 18615151533@163.com (W.Z.); 19833836160@163.com (Z.Y.); guorui4u@stu.hrbnu.edu.cn (R.G.); wangdan19881234@163.com (D.W.)

**Keywords:** abiotic stress, rice, aniline, physiology, multi-omics

## Abstract

The accumulation of aniline in the natural environment poses a potential threat to crops, and thus, investigating the effects of aniline on plants holds practical implications for agricultural engineering and its affiliated industries. This study combined physiological, transcriptomic, and metabolomic methods to investigate the growth status and molecular-level response mechanisms of rice under stress from varying concentrations of aniline. At a concentration of 1 mg/L, aniline exhibited a slight growth-promoting effect on rice. However, higher concentrations of aniline significantly inhibited rice growth and even caused notable damage to the rice seedlings. Physiological data indicated that under aniline stress, the membrane of rice underwent oxidative damage. Furthermore, when the concentration of aniline was excessively high, the cells suffered severe damage, resulting in the inhibition of antioxidant enzyme synthesis and activity. Transcriptomic and metabolomic analyses indicated that the phenylpropanoid biosynthesis pathway became quite active under aniline stress, with alterations in various enzymes and metabolites related to lignin synthesis. In addition to the phenylpropanoid biosynthesis pathway, amino acid metabolism, lipid metabolism, and purine metabolism were also critical pathways related to rice’s response to aniline stress. Significant changes occurred in the expression levels of multiple genes (e.g., PRX, C4H, GST, and ilvH, among others) associated with functions such as antioxidant activity, membrane remodeling, signal transduction, and nitrogen supply. Similarly, notable alterations were observed in the accumulation of various metabolites (for instance, glutamic acid, phosphatidic acid, phosphatidylglycerol, and asparagine, etc.) related to these functions. Our research findings have unveiled the potential of compounds such as phenylpropanoids and amino acids in assisting rice to cope with aniline stress. A more in-depth and detailed exploration of the specific mechanisms by which these substances function in the process of plant resistance to aniline stress (for instance, utilizing carbon-14 isotope tracing to monitor the metabolic pathway of aniline within plants) will facilitate the cultivation of plant varieties that are resistant to aniline. This will undoubtedly benefit activities such as ensuring food production and quality in aniline-contaminated environments, as well as utilizing plants for the remediation of aniline-polluted environments.

## 1. Introduction

Aniline (AN) is widely used as an intermediate in the industries of rubber, dyes, pesticides, spices, and varnish, etc. Due to its toxicity and persistence, AN has been classified by the EPA as an organic pollutant that needs to be monitored and controlled with priority [1]. Unfortunately, AN still enters ecosystems along with wastewater and gas during the production process and then accumulates. For instance, a concentration of aniline at 0.3 mg/L was detected within the aquifer located in the vicinity of a coal gasification processing facility [2]. Moreover, various accidents can also cause serious AN leakage: seven days after an aniline leakage incident occurred at a chemical plant, a concentration of aniline was measured at 72 mg/L in the aquatic environment located more than 80 km away from the site of the leakage [3].

The accumulation of AN inevitably poses a threat to various organisms. AN has diverse adverse effects on plant growth. Several investigations have revealed that AN induces non-stomatal limitations on photosynthesis in plants, inhibiting the light reactions within mesophyll cells and resulting in a decrease in net photosynthetic rate [4,5]. Consequently, this interference in the photosynthesis process hinders plant growth, and even introduces genotoxicity into plant root tip cells [6]. Moreover, AN stress can cause breaks or cross-linking in plant DNA chains, and decrease the DNA content in plant buds and roots [7]. Aniline can also exert detrimental impacts on human health. When absorbed, AN oxidizes hemoglobin in red blood cells and produces methemoglobin, which affects oxygen transportation [8]. Long-term exposure to AN significantly increases the risk of cancer [9]. Crops grown in aniline-contaminated environments exhibit accumulation of aniline within their tissues [4]. In such circumstances, even if the concentration of aniline in the crops is low, long-term consumption of these crops can lead to the aforementioned situation.

Involving various physiological functions such as gene regulation, transcription, translation, signal transduction, and energy metabolism, the metabolic process of plant response to toxin stress is rather complicated. Since genes and metabolites are major participants in plant growth and stress responses, transcriptome sequencing and metabolomics technologies that can quantitatively measure changes in gene expression and metabolites can provide researchers with a deeper perspective on the mechanisms of plant responses to toxin stress. Exploiting HRMAS NMR-based (High-Resolution Magic-Angle Spinning Nuclear Magnetic Resonance-based) metabolomics tools, Blondel et al. [10] obtained detailed information on changes in carbohydrates, amino acids, tricarboxylic acid cycle intermediates, and fatty acids in corn roots under organochlorine pesticide stress, through which conclusions surrounding an imbalance in gluconeogenesis/glycolysis, a new distribution of internal nitrogen compounds, and impairment of cell integrity and viability could be drawn; Pereira et al. [11] found changes in phenylalanine, polyphenols, fumaric acid, and other substances in lettuce under mancozeb stress. These changes indicate higher oxidative stress and up-regulation of the tricarboxylic acid cycle during growth. Metabolome analysis alone cannot explain the genetic mechanism that affects phenotypes, which can be exposed by transcriptional analysis. Using integrative metabolomics and transcriptomics, Chen et al. [12] investigated the molecular mechanisms underlying the different tolerance ability of two rice varieties to 2,2’, 4,4’-tetrabromodiphenyl ether (BDE-47) to understand the potential cause of the difference in tolerance. Liu et al. [13] discovered significant differences in the expression regulation and metabolic responses of key rice genes under the stress of three typical pesticides. As a highly stubborn organic pollutant [14], aniline’s harm to plants has been discovered and to some extent investigated [7]. However, the physiological and molecular mechanisms of its toxicity to plants are still unclear and require further exploration.

Rice (*Oryza sativa* L.), as a staple food source for approximately 4 billion people [15,16], is one of the most important food crops [17] and is frequently used as a model plant in molecular biology research [18]. Therefore, rice is a reasonable candidate for studying the toxicity of AN to plants at both physiological and molecular levels. The existing literature [4,5,6,7] provides us certain understanding of the growth toxicity and genotoxicity of AN; however, the impacts of AN on plant gene expression and metabolism have not yet been sufficiently explored. To the best of our knowledge, there is currently no literature available that utilizes omics tools to investigate how plants respond to aniline stress. Therefore, the present study aims to comprehensively investigate the responses of rice to different concentrations of aniline stress from both physiological and multi-omics perspectives.

Based on the research objectives, we propose the following research hypotheses:

1. Rice seedlings will exhibit distinct physiological responses under different concentrations of aniline stress, and these responses demonstrate a certain correlation with the concentration of aniline.

2. Different concentrations of aniline stress result in significant differences in gene expression and metabolite accumulation in rice, which may be associated with the stress resistance mechanisms of rice.

3. Through integrated transcriptome and metabolome analysis, we can identify key metabolic pathways involved in rice’s response to aniline stress, which may play pivotal roles in rice stress resistance.

Based on the aforementioned research hypotheses, the following work was conducted in this study: under different concentrations of AN stress, we measured variations in the contents of malondialdehyde (MDA), hydrogen peroxide (H_2_O_2_), soluble sugar, and soluble protein, as well as changes in the activities of three enzymes, superoxide dismutase (SOD), peroxidase (POD), and catalase (CAT), in rice. Based on transcriptomics and metabolomics tools, we identified differentially expressed genes, differential metabolites, and the key metabolic pathways involved in the AN stress response in rice. This study clarified the physiological and molecular mechanisms underlying the response of rice to aniline stress, which can serve as the basis for further research. Practically, the identification of the critical genetic and metabolic factors involved offers a foundation for the development of aniline-tolerant rice varieties through genetic engineering or marker-assisted selection. This could have profound impacts on crop production in areas contaminated with aniline, enabling farmers to continue cultivating rice without significant yield losses. Furthermore, our findings raise several intriguing questions for future investigation. For instance, further research could explore the regulatory networks and interactions among the identified genes and metabolites, providing deeper insights into the rice plant’s stress response mechanisms. Additionally, studies could investigate the potential for cross-tolerance among different stressors, examining whether the identified genes and pathways also confer tolerance to other environmental stressors.

## 2. Results

### 2.1. Changes in Physiological Indicators

**Figure 1:**
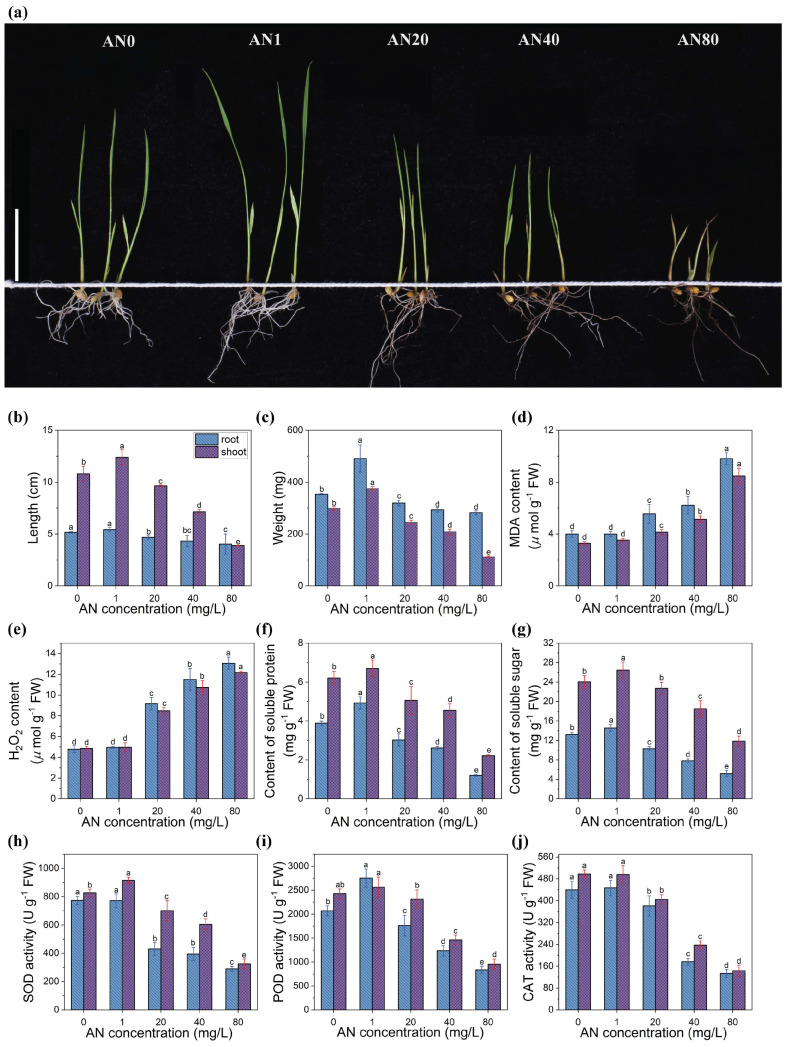
The effect of different concentrations of aniline on (**a**) the growth status of rice seedlings (the white bar on the left represents 5 cm), (**b**) the length of root and shoot, (**c**) the weight of root and shoot, and the effect on the content of (**d**) MDA, (**e**) H_2_O_2_, (**f**) soluble protein, (**g**) soluble sugar, and the activity of (**h**) SOD, (**i**) POD, and (**j**) CAT in root and shoot. ANOVA was utilized to compare the statistical differences among different groups, and lowercase letters indicates significant differences.

The status of the rice seedlings significantly differed when exposed to aniline solutions at different concentrations (Figure 1a). As the concentration of aniline increased, it became more obvious that leaves turned yellow and wilted, roots became black and thinner, and the growth of rice was inhibited.

In comparison to the control group, the length and weight of rice seedlings in Group AN1 (AN1 represents an aniline solution with a concentration of 1 mg/L, and so on) showed a slight increase. As the concentration of aniline continued to increase, the length and weight of the seedlings gradually decreased (Figure 1b,c). Compared to group AN0 (AN0 represents the control group, where 0 indicates zero aniline content), the root length and shoot length of seedlings in group AN80 decreased by approximately 22.03% and 63.91%, respectively, while the root weight and shoot weight decreased by about 20.22% and 62.61%, respectively.

In the AN20, AN40, and AN80 groups, the MDA and H_2_O_2_ contents in both the root and shoot of rice seedlings significantly rose with an increase in aniline concentration (Figure 1d,e). Compared to the AN0 group, the MDA content in the root and shoot of rice seedlings in the AN80 group increased by approximately 145.3% and 159.2%, respectively, and the H_2_O_2_ content increased by about 173.2% and 150.1%, respectively.

In the AN1 group, the soluble protein (SP) content in root and shoot increased by approximately 25.64% and 8.06%, respectively, compared to the AN0 group. When the aniline concentration increased from 1 mg/L to 20 mg/L, the SP content in root and shoot decreased by 38.78% and 23.88%, respectively. With further increases in aniline concentration, the SP content continued to decline. Compared to the peak values observed in the AN1 group, the SP content in root and shoot in the AN80 group decreased by 75.51% and 67.16%, respectively (Figure 1f). The trends in the soluble sugar content (Figure 1g) and the activities of the three antioxidant enzymes SOD (Figure 1h), POD (Figure 1i), and CAT (Figure 1j) were quite similar to those of SP: peak values were observed in the AN1 group, followed by continuous decreases in soluble sugar content and enzyme activities in the AN20, AN40, and AN80 treatment groups.

### 2.2. Transcriptomic Analysis

#### 2.2.1. Transcriptome Sequencing and DEG Identification

A total of 69.66 GB of QC (quality-controlled) data were obtained. After filtering, the Q30 for all samples exceeded 94.22%, the GC (guanine and cytosine) content ranged from 52.80% to 54.67%, and the error rates were below 0.03% (Table S1). The portion of uniquely mapped reads was above 90.88% (Table S2), affirming that the sequencing data were qualified.

**Figure 2:**
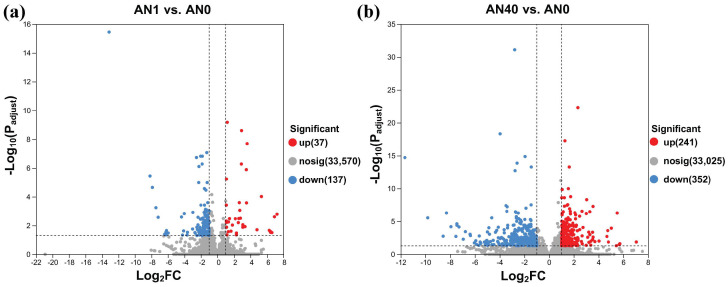
Up-regulated (red solid circles, denoted as “up”), down-regulated (blue solid circles, denoted as “down”) and non-significant (gray solid circles, denoted as “nosig”) genes identified in the (**a**) AN1 and (**b**) AN40 groups, respectively, compared to the AN0 group.

Comparative analysis between the AN0 and AN1 groups, as well as between the AN0 and AN40 groups, was performed. As can be seen in Figure 2a,b, 174 (37 up-regulated and 137 down-regulated) and 593 (241 up-regulated and 352 down-regulated) differentially expressed genes (DEGs) were, respectively, identified in the pairwise comparisons of AN1 vs. AN0 and AN40 vs. AN0. Detailed information on the DEGs of the two comparison groups are given in Tables S3 and S4, respectively.

#### 2.2.2. GO and KEGG Pathway Analysis

**Figure 3:**
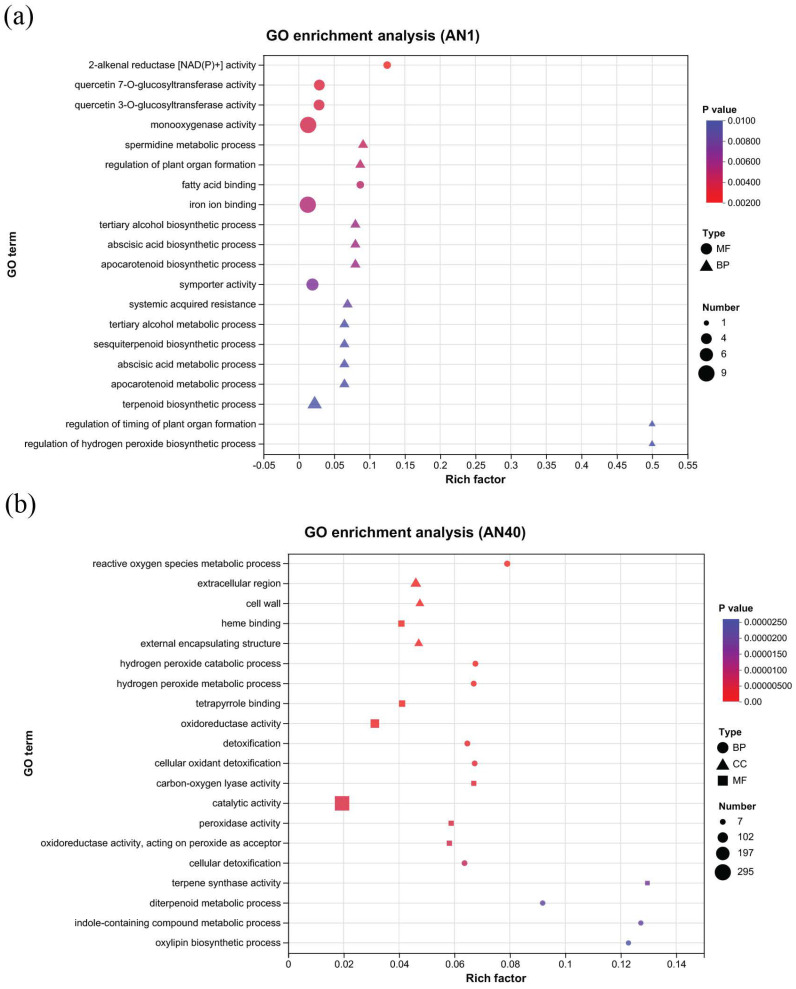
The top 20 terms with the highest enrichment significance in the (**a**) AN1 and (**b**) AN40 groups.

We performed functional classification of the differential genes identified in the two experimental groups. The top 20 GO (gene ontology) terms with the highest enrichment significance in groups AN1 and AN40 are shown in Figure 3a,b, respectively. In both the AN1 and AN40 groups, a substantial number of DEGs were associated with the terms of “integral component of membrane” and “membrane” within the cellular component ontology, and “transferase activity” and “oxidoreductase activity” within the molecular function ontology. Please refer to Tables S5 and S6 for more detailed GO annotations of the DEGs in the AN1 and AN40 groups.

**Figure 4:**
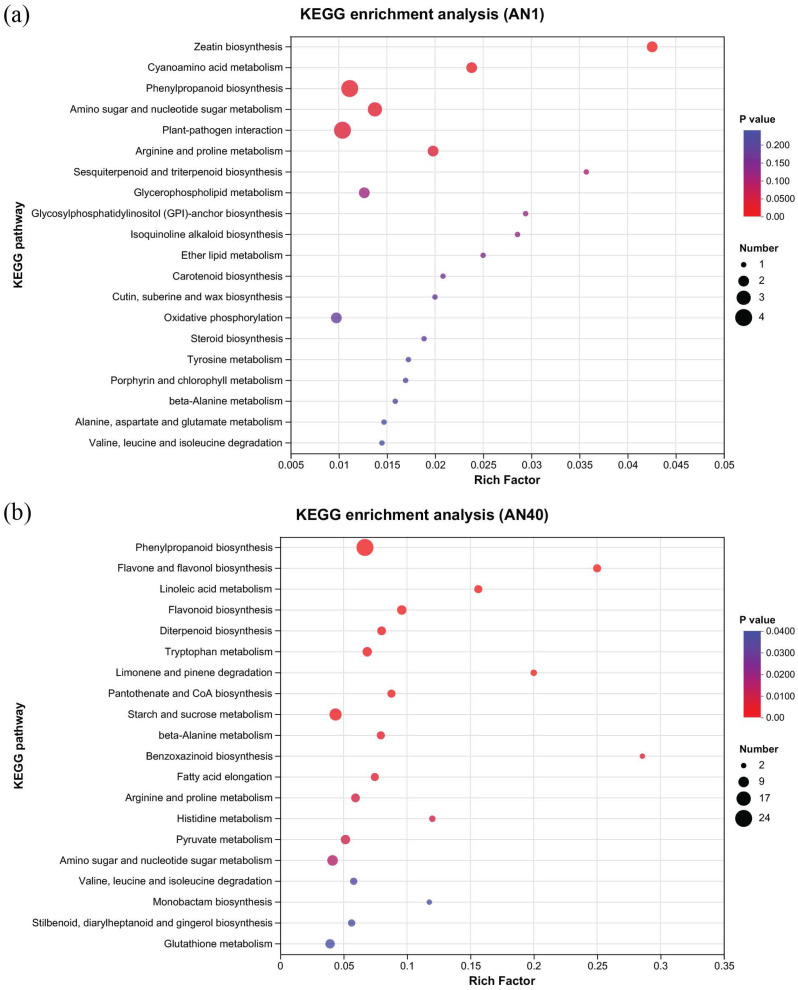
The top 20 pathways ranked by enrichment significance levels in the (**a**) AN1 and (**b**) AN40 groups.

KEGG (Kyoto Encyclopedia of Genes and Genomes) pathway enrichment analysis was performed on DEGs to investigate the key metabolic pathways involved in rice development in response to aniline stress. Figure 4 presents the top 20 pathways ranked by enrichment significance levels in the AN1 (Figure 4a) and AN40 (Figure 4b) groups. In both the AN1 and AN40 groups, we observed enrichment of DEGs in the phenylpropanoid biosynthesis pathway and amino acid metabolism-related pathways, as well as the amino sugar and nucleotide sugar metabolism pathways. Additionally, in the AN40 group, DEGs were also found enriched in pathways such as glutathione metabolism, starch, and sucrose metabolism.

#### 2.2.3. qRT-PCR Verification of Gene Expression

**Figure 5:**
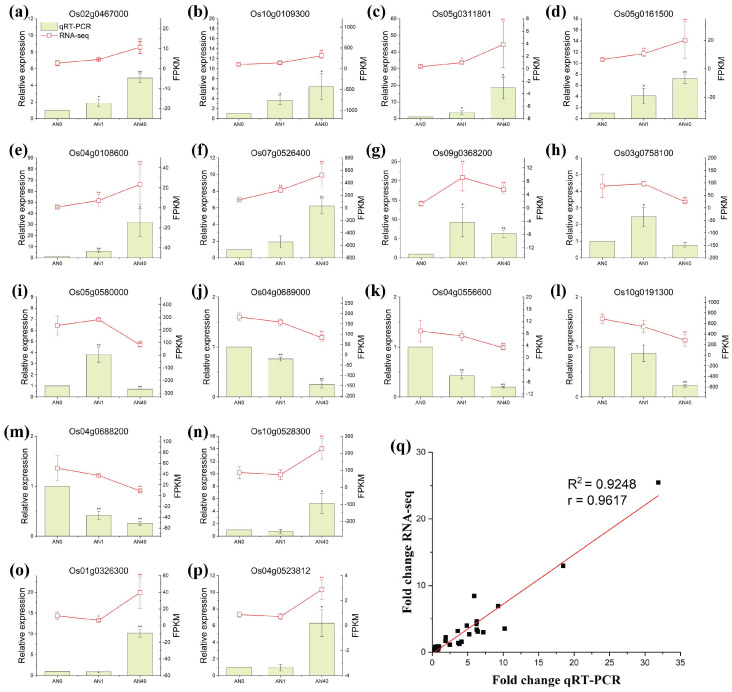
Results of qRT-PCR verification. From (**a**–**p**): comparison between the relative expression measured by qRT-PCR (green bars corresponding to the left Y-axis) and the expression levels obtained by RNA-seq (red symbolled lines corresponding to the right Y-axis) of the randomly selected 16 DEGs. The asterisks “*” and “**” indicate *p*-values less than 0.05 and 0.01, respectively. (**q**): fitting and correlation between the qRT-PCR and RNA-seq results.

We randomly selected 16 DEGs for qRT-PCR (Quantitative Real-time Polymerase Chain Reaction) verification. As shown in Figure 5, the qRT-PCR results for the 16 randomly selected genes were completely consistent with the trends observed in the RNA-seq data across different treatment groups, i.e., the qRT-PCR and RNA-seq data strongly correlated, indicating that the RNA-seq data were reliable. Table S7 provides the information on the randomly selected genes and the primers used. Additionally, Table S8 presents the raw data and statistical metrics for qRT-PCR and RNA-seq, enabling readers to obtain more detailed information regarding the results of both qRT-PCR and RNA-seq.

### 2.3. Metabolomic Analysis

#### 2.3.1. Quality Assessment of Metabolomic Data

**Figure 6:**
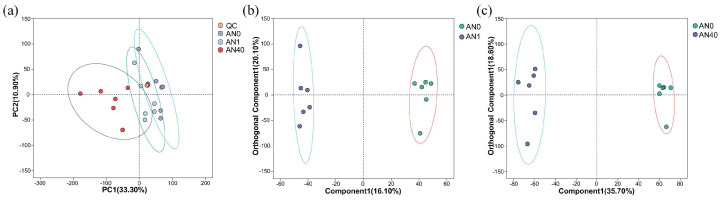
(**a**) PCA and OPLS-DA for (**b**) AN1 vs. AN0 and (**c**) AN40 vs. AN0 of the metabolomic data.

As can be seen in Figure 6a, the data points corresponding to the QC samples were tightly clustered among those of the control and treatment groups, signifying a stable and consistent data collection process, and the close alignment suggests that the QC samples effectively captured the overall characteristics of the control and treatment samples. The results of the OPLS-DA (Orthogonal Partial Least Squares-Discriminant Analysis) for AN1 vs. AN0 (Figure 6b) and AN40 vs. AN0 (Figure 6c) indicate that the data for both the AN1 and AN40 groups can be well-separated from that of the AN0 group.

#### 2.3.2. Identification and Analysis of DAMs

**Figure 7:**
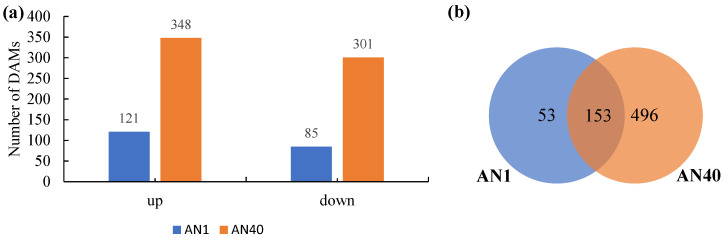
(**a**) Number of up-regulated and down-regulated DAMs identified in the AN1 and AN40 groups, respectively. (**b**) Venn diagram of DAM sets corresponding to the AN1 and AN40 groups.

As shown in Figure 7a, 206 (121 up-regulated and 85 down-regulated) and 649 (348 up-regulated and 301 down-regulated) DAMs (differentially accumulated Mmetabolites) were identified in the AN1 and AN40 groups, respectively. Additionally, there were 153 common DAMs between the two groups (Figure 7b).

**Figure 8:**
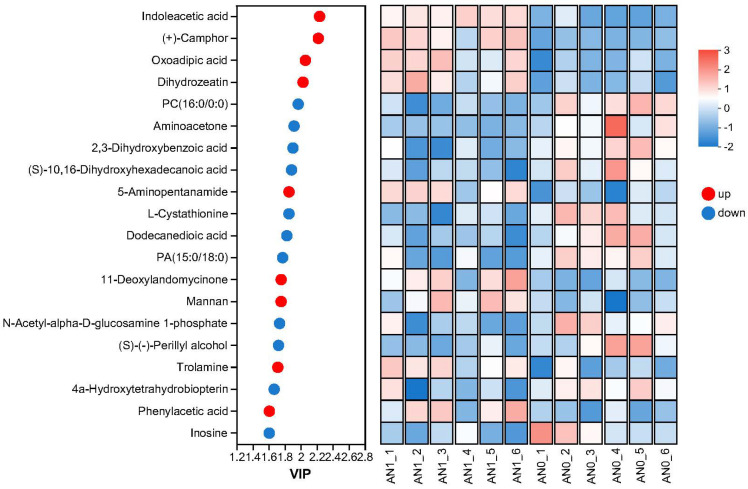
The top 20 DAMs with the highest VIP scores in the AN1 group and their relative expression levels in each sample.

**Figure 9:**
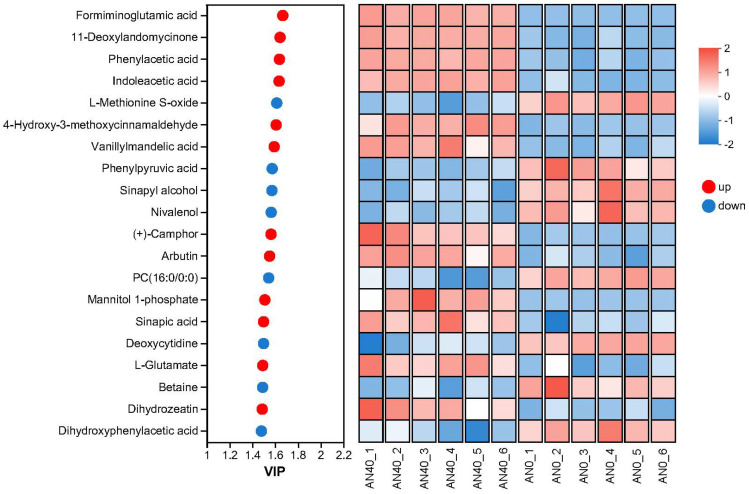
The top 20 DAMs with the highest VIP scores in the AN40 group and their relative expression levels in each sample.

Figure 8 and Figure 9 present the top 20 DAMs ranked by VIP (Variable Importance in the Projection) scores in the AN1 and AN40 groups, respectively, along with their relative expression levels in each sample. These DAMs were primarily composed of five types of compounds: benzenoids, lipids and lipid-like molecules, organic acids and derivatives, organic oxygen compounds, and organ heterocyclic compounds. For detailed data on DAMs in the AN1 and AN40 groups, please refer to Tables S9 and S10, respectively.

**Figure 10:**
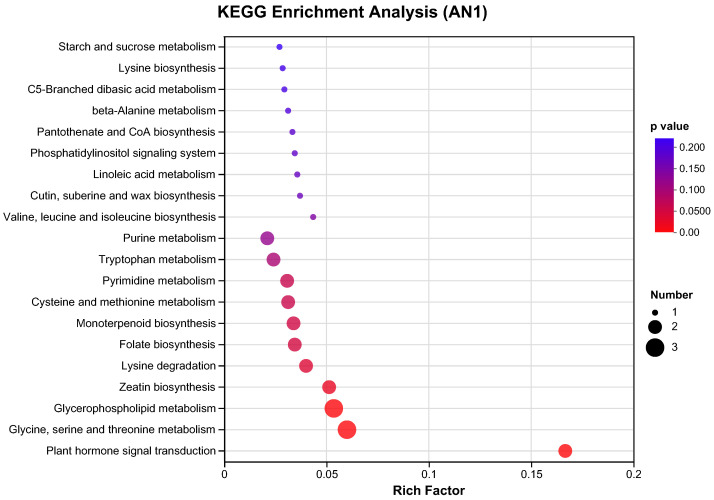
The top 20 pathways with the highest enrichment significance in the AN1 group.

**Figure 11:**
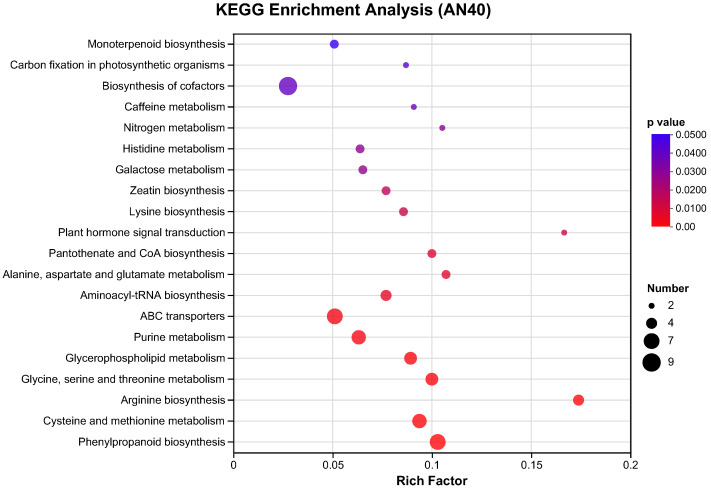
The top 20 pathways with the highest enrichment significance in the AN40 group.

The pathways of plant hormone signal transduction, glycine, serine, and threonine metabolism, glycerophospholipid metabolism, zeatin biosynthesis, lysine degradation, folate biosynthesis, and monoterpenoid biosynthesis were significantly enriched in the AN1 group (Figure 10). In contrast, more pathways were disturbed in the AN40 group (Figure 11). Phenylpropanoid biosynthesis, cysteine and methionine metabolism, and arginine biosynthesis were the most significantly enriched pathways in the AN40 group. Additionally, pathways significantly enriched in the AN1 group (with the only exception being the folate biosynthesis pathway) were also significantly enriched in the AN40 group. Additionally, more DAMs were involved in each enriched pathway in the AN40 group.

**Figure 12:**
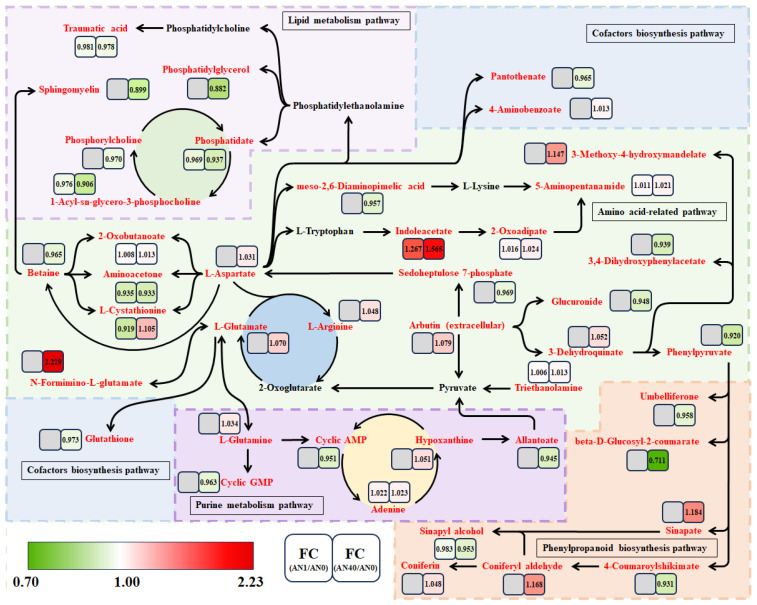
The DAM pathway network of rice under aniline stress. The gray tiles without numerical labels indicate no significant difference.

We constructed a pathway network using the KEGG metabolic pathway database to systematically illustrate the changes in the DAMs under aniline stress and their possible associations (Figure 12). To keep the simplicity and clarity of the illustration, the network does not include all of the DAMs in the AN1 and AN40 groups. Instead, we focused on the following five modules: amino acid-related pathways, phenylpropanoid biosynthesis pathways, purine metabolism pathways, lipid metabolism pathways, and cofactor biosynthesis pathways.

### 2.4. Integrated Analysis of Transcriptomics and Metabolomics

**Figure 13:**
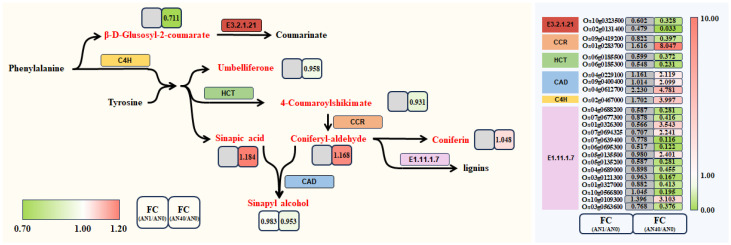
The DEGs and DAMs identified in the phenylpropanoid biosynthesis pathway and their relative expression levels.

Transcriptomics and metabolomics combined analysis indicated that the phenylpropanoid pathway was quite active in rice’s response to aniline stress. Under 40 mg/L aniline stress, 24 DEGs and 7 DAMs were differentially expressed in this pathway. In the AN1 group, the majority of genes and metabolites did not undergo significant changes. In contrast, in the AN40 group, sinapic acid, coniferyl-aldehyde, and coniferin accumulated, while sinapyl alcohol and β-D-glucosyl-2-coumarate decreased. Among the identified DEGs in this pathway, C4H (cinnamate 4-hydroxylase) and CAD (cinnamylalcohol dehydrogenase) were up-regulated, HCT (Hydroxycinnamoyl-CoA shikimate/quinate hydroxycinnamoyltransferase) was down-regulated, and *Os09g0419200* and *Os01g0283700*, which are members of CCR (Cinnamoyl-CoA reductase), were down- and up-regulated, respectively. Most members with EC code E1.11.1.7 were down-regulated. Figure 13 shows the relative expression levels of these DEGs and DAMs.

## 3. Discussion

In our study, we treated rice seedlings with various concentrations of aniline and utilized physiological, transcriptomic, and metabolomic approaches to investigate the physiological and molecular mechanisms by which rice responds to aniline stress, thereby providing a deeper insight into how plants respond to organic pollutant stress.

### 3.1. Physiological Responses of Rice to Aniline Stress

The variations observed in MDA and H_2_O_2_ in our experiments were highly consistent with those reported by Tao et al. [7]. Serving as important signaling molecules, reactive oxygen species (ROS) are produced not only under stress conditions but also during normal physiological processes. Maintaining an appropriate cellular ROS level, i.e., redox homeostasis, is beneficial to biological systems [19]. In our study, a low concentration of aniline (1 mg/L) stimulated the production of a small amount of ROS in rice, resulting in increased plant height and weight under this concentration. However, high concentrations of aniline (20 mg/L and above) led to excessively high levels of ROS in rice, disrupting redox homeostasis and causing oxidative damage to rice cells. This damage manifested as narrowed leaves, reduced plant height, and tip chlorosis, and became more severe as the aniline concentration increased. With the increase in aniline concentration, the levels of MDA and H_2_O_2_ in the rice increased, indicating that aniline stress led to an elevation in ROS and oxidative damage to rice cells. Plants activate antioxidant enzymes, such as SOD, POD, and CAT, to counteract oxidative damage, eliminate free radicals, and neutralize toxic oxidative intermediates, thereby maintaining internal balance [20]. Our research indicates that with increasing aniline concentration, the activity of SOD, POD, and CAT first rose and then fell. Under low concentration of aniline stress, rice can effectively eliminate ROS by increasing the activity of antioxidant enzymes, thereby reducing oxidative damage. However, when the concentration of aniline exceeded a certain threshold, cells suffered damage, resulting in the plant’s inability to maintain sufficient regulatory capacity for antioxidant enzyme activity. Consequently, this led to the accumulation of ROS, a decrease in antioxidant enzyme activity, and even enzyme inactivation.

Soluble sugars and soluble proteins can regulate life activities under stress conditions, thereby influencing plant’s stress tolerance [21,22]. In this study, the levels of soluble sugars and proteins showed a trend of first increasing and then decreasing, which was consistent with the observations of Jiang et al. [23]. Under a low concentration of aniline stress (1 mg/L in our study), rice seedlings cope with environmental pressure by enhancing photosynthesis and respiration, which leads to an increase in the synthesis of soluble sugars and proteins while activating the antioxidant defense system to eliminate ROS [24,25]. On the other hand, in low concentrations of aniline solution, plant cells experience a certain level of osmotic stress. To resist this stress, cells increase the production of soluble sugars to elevate the osmotic pressure inside, helping to maintain cellular water balance and turgor, which also results in an increase in soluble sugars [24]. However, when the concentration of aniline reaches a certain threshold, the oxidative stress faced by the plants significantly increases, leading to greater cellular damage and higher energy consumption. Cells prioritize the consumption of stored sugars and proteins to address urgent energy needs [26], resulting in a decrease in the levels of soluble sugars and proteins. Furthermore, high concentrations of aniline may impair the roots’ ability to absorb and utilize nitrogen, inhibit protein synthesis, and concurrently, excessive stress on cells may lead to apoptosis and dysfunction, further suppressing the synthesis of soluble sugars and proteins, and ultimately reducing their total content. This process reflects the complex physiological responses of plants to environmental stress, including active adaptive measures and metabolic dysregulation triggered by severe stress.

### 3.2. The Phenylpropanoid Biosynthesis Pathway Responded to Aniline Stress

The phenylpropanoid biosynthesis pathway is involved in the synthesis of lignin and various plant secondary metabolites, enhancing plant stress resistance and serving as a critical node in plant metabolic regulation. In this study, compared to other pathways, the phenylpropanoid biosynthesis pathway exhibited a relatively active response to aniline stress.

C4H, a cytochrome P450-dependent monooxygenase, catalyzes the conversion of trans-cinnamic acid to p-coumaric acid, which is a critical step in the biosynthesis of secondary metabolites such as lignin and flavonoids. In this study, the gene *Os02g0467000* (please refer to Table S4, and the same for the specific genes discussed below), which encodes C4H, was up-regulated by 3.997-fold under aniline stress at a concentration of 40 mg/L, being consistent with the findings of Kim et al. [27]. Under stressful conditions, rice enhances its antioxidant capacity and stabilizes its cellular structures by up-regulating C4H, thereby bolstering stress resistance.

HCT is responsible for transferring hydroxycinnamoyl groups from CoA (coenzyme A) esters to shikimate or quinate, serving as a crucial functional enzyme in lignin biosynthesis [28]. In this study, aniline stress inhibited the expression of HCT, with two genes (*Os06g0185500* and *Os06g0185300*) encoding HCT being down-regulated to 0.37-fold and 0.23-fold, respectively. Consequently, the content of 4-coumaroyl shikimate, which is produced from p-coumaroyl CoA under the catalysis of HCT enzyme, was also down-regulated.

CCR catalyzes reductive reactions to synthesize p-coumaraldehyde, caffealdehyde, coniferyl aldehyde, and sinapaldehyde [29]. Studies have suggested that CCR may play a pivotal role in rate control within the regulation of lignin biosynthesis [30]. In our experimental results, the genes *Os09g0419200* and *Os01g0283700*, which encode CCR, were down- and up-regulated, respectively. This differential expression suggests that these two CCR genes play distinct roles in rice’s response to aniline stress. Concurrently, we also observed an up-regulation in the content of coniferyl aldehyde, a key downstream intermediate of CCR in the lignin biosynthesis pathway. The increase in coniferyl aldehyde content may be due to *Os01g0283700* being more active or specific in producing coniferyl aldehyde or its precursors, or it may represent a compensatory response to the differential expression of CCR genes, indicating that rice may be attempting to redirect metabolic resources towards lignin production or other phenolic pathways to enhance stress tolerance.

Under aniline stress, both sinapic acid and coniferyl aldehyde, which are related to the synthesis of sinapyl alcohol, showed increased levels, accompanied by an up-regulation of CAD gene expression. However, the content of sinapyl alcohol decreased, revealing the complex regulatory mechanisms of lignin metabolism in rice in response to aniline stress. As important precursors in the lignin biosynthesis pathway, the increase in sinapic acid and coniferyl aldehyde usually indicates enhanced lignin synthesis activity. Especially, coniferyl aldehyde, as a substrate for CAD enzyme, its elevated content suggests sufficient substrate availability for CAD-catalyzed conversion to sinapyl alcohol. Contrary to this, however, the content of sinapyl alcohol did not increase accordingly but rather showed a downward trend. This indicates that under aniline stress, some regulatory changes may have occurred in the lignin metabolism pathway of rice, affecting the synthesis of sinapyl alcohol. Up-regulation of CAD expression usually accelerates the conversion of coniferyl aldehyde to sinapyl alcohol. However, in this study, the up-regulation of CAD expression failed to reverse the decreasing trend of sinapyl alcohol content. This may be due to constraints on CAD enzyme activity from other factors, such as post-translational modifications of enzyme proteins [31], cofactor availability limitations [32], or changes in the activity of other key enzymes in the metabolic pathway.

As a phenolic compound, β-D-Glucosyl-2-coumarate possesses the ability to scavenge or neutralize free radicals within plant tissues, thereby exerting antioxidant effects [33]. Research conducted by Mashiane et al. [34] has shown that β-D-Glucosyl-2-coumarate is associated with the antioxidant capacity of plants. Additionally, Esposito et al. [35] discovered that β-D-Glucosyl-2-coumarate, as a glucoside derivative of coumarin, exhibits mild toxicity to plants and inhibits plant development. In the present study, the accumulation of β-D-Glucosyl-2-coumarate in the AN40 group was down-regulated to 0.711 times its normal level. This may represent a unique regulatory mechanism in rice, in response to the combined effect of oxidative stress and the inherent toxicity of β-D-Glucosyl-2-coumarate.

Additionally, under 40 mg/L aniline stress, we observed differential expression of fourteen genes with EC number EC: 1.11.1.7 in the phenylpropanoid pathway, among which only three were up-regulated. Notably, the genes encoding peroxidase (PRX) were largely suppressed, leading to a decreased ability of rice to scavenge ROS. The class III PRX gene family is a plant-specific member of the peroxidase superfamily, involved in regulating POD activity, scavenging ROS, and closely related to various physiological processes such as cell growth, lignification, and abiotic stress responses [36]. Among the eleven differentially expressed PRX genes identified in our study, nine (prx14, prx34, prx49, prx60, prx64, prx69, prx92, prx108 and prx129) were significantly down-regulated, indicating that the expression of PRXs was inhibited under 40 mg/L aniline stress. This suppression disrupted the ability to regulate POD activity and ROS scavenging, suggesting that the accumulation of ROS exceeded the tolerance threshold of rice seedlings. Both aniline and ROS may contribute to the disruption of the integrity of the cellular membrane system and the inhibition of protein function.

### 3.3. The Amino Acid Metabolism Pathways Responded to Aniline Stress

In addition to their role in protein synthesis, amino acids function as intermediates in the TCA (tricarboxylic acid) cycle for energy generation and accumulate as antioxidants in plants subjected to abiotic stress [37]. Our research revealed alterations in specific genes and metabolites involved in amino acid metabolism under aniline stress, indicating that rice mounts relevant defense responses by modulating amino acid metabolism and other metabolic pathways.

The up-regulation and accumulation of asparagine under aniline stress could be attributed to its pivotal role in nitrogen storage and reutilization. During stressful conditions, plants elevate their nitrogen demand to synthesize additional stress-resistance-related compounds, including antioxidant enzymes and osmoregulatory substances. As a significant intermediate in nitrogen metabolism, the augmented accumulation of asparagine fulfills the plant’s nitrogen requirement, thereby facilitating the activation and sustenance of stress resistance mechanisms [38]. Furthermore, asparagine may indirectly bolster the plant’s stress tolerance by stimulating root growth and nutrient uptake.

The accumulation of glutamic acid is intimately linked to its pivotal role in antioxidant defense mechanisms. As a fundamental constituent of the antioxidant defense system, the elevated accumulation of glutamic acid boosts the activity of antioxidant enzymes within the plant, thereby facilitating more efficient scavenging of ROS and alleviating oxidative damage. Glutamic acid also influences the antioxidant capacity of plants through another pathway. As one of the precursors for glutathione synthesis, glutamic acid undergoes a series of enzymatic reactions to convert into glutathione. Studies by Huang et al. [39] have demonstrated that exogenous application of glutamic acid results in an increase in the contents of ascorbic acid (AsA) and glutathione (GSH), as well as an enhancement in the activities of enzymes such as monodehydroascorbate reductase within the ascorbate-glutathione cycle (AsA-GSH cycle). Consequently, the accumulation of glutamic acid indirectly elevates the antioxidant capacity of the AsA-GSH cycle, thereby improving the stress tolerance of plants. Furthermore, glutamic acid is involved in osmotic regulation by adjusting intracellular osmotic pressure to counteract water stress induced by stressful conditions. Studies conducted by Gill et al. [40] have demonstrated that increased glutamic acid accumulation can stimulate the expression of glutathione S-transferase (GST) genes, which is consistent with the experimental findings of the present study (up-regulation of *Os10g0527601*, *Os01g0369800*, *Os10g0525500*, *Os10g0528300*, and *Os10g0528100*). The up-regulation of GST gene expression enhances the plant’s antioxidant capacity, aiding its response to abiotic stress, minimizing ROS accumulation, and maintaining intracellular redox balance. Additionally, GST genes assist in plant detoxification by catalyzing the conjugation of glutathione (GSH) with harmful compounds, thereby enhancing their stress tolerance [41]. The observed decrease in glutathione accumulation in the AN40 group may be attributable to this effect.

The up-regulation and accumulation of arginine in response to aniline stress can be attributed to its crucial role in polyamine synthesis and signaling [42]. Polyamines possess a wide array of biological activities and are crucial for processes such as cell division, cell elongation, and stress tolerance. As a precursor in polyamine synthesis, the elevated accumulation of arginine promotes the synthesis of polyamines, thereby enhancing the plant’s stress resistance mechanisms. Furthermore, arginine may function as a signaling molecule in the transduction of stress signals, responding to aniline stress by modulating the expression of related genes.

Tryptophan serves as the primary precursor for the synthesis of indole-3-acetic acid (IAA), with acetaldehyde dehydrogenase (ALDH) catalyzing the formation of IAA. Under stressful conditions, the increased content of IAA plays a positive role in plant growth [43], which may be one of the main reasons for the promoted growth of rice in the AN1 group. Ke et al. found that rice ALDH2B1 is a key regulator of growth and defense in rice [44]. Gao et al. observed that the expression levels of some ALDH genes were up-regulated under drought, high salt stress, and ABA treatment, suggesting that the products of these genes may play a role in the adaptation of rice to osmotic stress [45]. In this study, genes encoding ALDH (*Os02g0730075*, *Os12g0178000*, and *Os02g0730000*) were up-regulated, catalyzing the synthesis of IAA and 3-IAA, which may represent a mechanism for rice to cope with aniline stress. We also observed a down-regulation in the expression of genes encoding YUCCA, specifically *Os07g0437000* and *Os04g0128900*, suggesting that rice may regulate the expression of YUCCA genes through a negative feedback loop mediated by the SCFTIR1/AFB signaling pathway. This feedback regulation mechanism aids in maintaining a steady state of IAA levels within the plant. Our findings are consistent with the research results reported by Takato et al. [46].

A study by Liu et al. [47] found that the accumulation of 2-oxobutanoate can significantly up-regulate the expression of the livH and ilvE genes. In our experiments, with increased accumulation of 2-oxobutanoate, the expression of the ilvH gene (*Os11g0256000*) increased to 6.4 times that of the control group. The livH gene is involved in amino acid transport and metabolism, and its up-regulated expression can enhance the metabolic capacity of plants. In contrast, the ilvE gene (*Os03g0106400*) was not up-regulated, but rather down-regulated to 0.477 times its original expression, which may represent a unique defense mechanism in rice.

It is noteworthy that Teimuraz et al. [48] traced the metabolic process of aniline in plants using carbon-14 isotope-labeled aniline. They identified o-aminophenol as the major transformation product of aniline in plants and pointed out that the degradation rate of aniline in plants could be as low as 0.3%. In fact, in our study, we failed to detect the presence of o-aminophenol. Therefore, our view aligns with that of Teimuraz et al. [48]: the ability of rice to absorb and transform aniline is quite limited, and the damage caused by aniline to rice is mainly oxidative damage.

### 3.4. The Lipid and Puring Metabolism Pathways Responded to Aniline Stress

Under 40 mg/L aniline stress, we observed differential metabolites in metabolic pathways such as glycerophospholipids, glycerides, and alpha-linolenic acid. This indicates that rice may promote the production of signaling factors by regulating lipid content, thereby activating metabolic pathways involved in aniline stress resistance and enhancing the antioxidant activity of rice, reducing the toxicity of aniline in rice. Increasing evidence suggests that lipids are not only key components of cell membranes and energy suppliers for organisms, but also play significant roles in seed germination, organ differentiation, signaling, and responses to both biotic and abiotic stresses [49].

Phosphatidic acid (PA), phosphatidylglycerol (PG), and phosphatidylcholine (PC) in the glycerophospholipid metabolic pathway were down-regulated, which may have led to significant restructuring of the cell membrane structure. Glycerophospholipids are key components of the cell membrane bilayer, and their quantity and composition directly affects membrane fluidity and function. The down-regulation of PA, PG, and PC may have reduced membrane fluidity, making the membrane structure more rigid, which in turn affected the normal function and distribution of membrane proteins [50].

PA and sphingomyelin, as signaling molecules, can regulate the activity of various signaling pathways, including cell proliferation and stress responses [51]. A decrease in their concentrations may lead to impaired signal transduction, affecting the cell’s ability to perceive and respond to external stimuli. Additionally, a reduction in PG and PC may disrupt membrane-associated signaling pathways, lowering the activity and function of membrane proteins, and further weakening the cell’s signaling capacity.

The elevation of MDA is a marker of lipid peroxidation, indicating an increased level of oxidative stress within a cell. In this case, the cell may reduce oxidative stressors by down-regulating the synthesis of glycerophospholipids while simultaneously activating antioxidant defense mechanisms, such as the activation of antioxidant enzymes (e.g., superoxide dismutase and catalase) and increasing the synthesis of antioxidant substances (e.g., glutathione) to cope with oxidative stress [52]. Therefore, the down-regulation of glycerophospholipids and the elevation of MDA together reflected the cell’s efforts to maintain redox balance through the regulation of antioxidant responses under aniline stress. Under 40 mg/L aniline stress, the accumulation of glutamine increased, which is consistent with the findings of Ulukapi and Nasircilar [53]. Under abiotic stress conditions, plants accumulate more glutamine to facilitate nitrogen metabolism, thereby obtaining more nitrogen for the synthesis of proteins and other essential metabolites to cope with the stressful environment.

Liu et al. [54] found that the accumulation of adenine increases under stress conditions, which is consistent with our experimental results. Under abiotic stress, the elevation of adenine may serve to enhance signaling efficiency, aiding plants in better perceiving and responding to stress, while also contributing to maintaining energy supply and ensuring normal physiological functions of plants under adverse conditions. In a 1 mg/L aniline solution, the growth of rice was promoted, possibly related to the increase in adenine.

The research results of Duszyn et al. [55] indicate that under abiotic stress, the activity of key enzymes in the signaling pathways of plants, such as adenylate cyclase (AC) and guanylate cyclase (GC), decreases, leading to a reduction in the production of cGMP (cyclic guanosine monophosphate) and cAMP (cyclic adenosine monophosphate). Our research findings are consistent with those of Duszyn et al. [55], with the accumulation levels of cGMP and cAMP decreasing to 0.963-fold and 0.951-fold, respectively. As second messengers, the decrease in cGMP and cAMP may impair signaling pathways, affecting the plant’s perception of and response to abiotic stress.

## 4. Materials and Methods

### 4.1. Reagents and Rice Cultivation

AN solutions at different concentrations were obtained by diluting aniline reagent (purchased from Tianli Chemical Reagent Co., Ltd., Tianjin, China) with sterile water.

In our experiments, we utilized five distinct concentrations of aniline solutions: 0 mg/L (serving as a control), 1 mg/L (to assess potential non-toxic stimulatory effects), and 20, 40, and 80 mg/L (representing a gradually increasing gradient of stress). The selection of these concentrations was based on a combination of previous research findings and our preliminary dose–response analysis. Firstly, a literature review was conducted to establish a broad range of aniline concentrations previously reported to elicit significant physiological responses in rice or related plant species, potentially including sublethal and toxic concentrations. Subsequently, a more extensive range of aniline concentrations (including those ultimately not selected for the main experiment) was used in a preliminary dose–response study to identify concentrations causing clear and reproducible changes in rice growth, metabolism, or gene expression, within a range allowing for meaningful comparisons and interpretations. The concentration of 1 mg/L was included as a near-control to assess any non-toxic stimulatory effects of aniline at very low levels. Concentrations of 20, 40, and 80 mg/L were chosen to represent a gradually increasing stress gradient. By selecting these concentrations, we aimed to comprehensively capture the range of rice responses to aniline stress, from potential stimulatory effects at very low concentrations to severe inhibitory effects at higher concentrations. This approach enabled a comprehensive assessment of aniline’s toxic threshold and dose-response relationship in rice, contributing to a deeper understanding of rice stress response mechanisms.

We used Longdao 5, which is a widely grown rice variety, in this study. The seeds used in the experiment all developed well and were surface-disinfected in 5% sodium hypochlorite (NaClO) solution [56] and then thoroughly rinsed with sterile water. The seeds were germinated in petri dishes under dark environment (7 d). Healthy seedlings with a shoot length of 3 ± 0.5 cm were collected and randomly assigned to five groups, with 30 seedlings in each group. The seedling groups were then moved into glass jars containing 40 mL aniline solutions at concentrations of 1, 20, 40, and 80 mg/L (denoted as AN1, AN20, AN40, and AN80 experimental groups) and 40 mL sterile water (denoted as control group, or AN0 for simiplicity), respectively. Both the experimental group and the control group were cultured at a constant temperature of 28 °C and a light cycle of 16:8 (light:dark) h with a light intensity of 250 $µ\mathrm{mol}\cdot m^{-2}\cdot s^{-1}$ for 10 days. The AN solutions and sterile water were renewed every day.

On the 11th day, the rice seedlings were collected and quickly frozen with liquid nitrogen (30 min), and then stored in an ultra-low temperature refrigerator at −80 °C.

### 4.2. Determination of Physiological Indicators

#### 4.2.1. Determination of MDA Content

We used an MDA content assay kit (AKFA013C, Boxbio Science & Technology Co., Ltd., Beijing, China) to determine the content of MDA (malondialdehyde, one of the products of cellular membrane lipid peroxidation, which serves as an indicator of oxidative stress levels in organisms). Following the user manual, the plant sample (0.1 g) was firstly thoroughly homogenized in the extraction solution (1 mL) on ice. After centrifuging (4 °C, 8000× *g*, 10 min), the supernatant was collected and mixed with the reaction solution. The mixture was heated in a water bath (95 °C, 60 min) and then cooled to room temperature in an ice bath. After another centrifugation (room temperature, 10,000× *g*, 10 min), the supernatant was collected. We measured the absorbance at 450 nm, 532 nm, and 600 nm. All the physiological experiments were run in triplicate.

#### 4.2.2. Determination of H_2_O_2_ Content

The content of H_2_O_2_ was determined using an H_2_O_2_ content assay kit (AKAO009C, Boxbio Science & Technology Co., Ltd., Beijing, China), according to the instructions in the manual. The absorbance was measured at 415 nm. For the sake of brevity, we do not list details of the assay procedure here and in Section 4.2.3.

#### 4.2.3. Determination of Antioxidant Enzyme Activity

The activity of SOD, POD, and CAT were determined using the corresponding activity assay kits (AKAO001C, AKAO005C, AKAO003-2C, Boxbio Science & Technology Co., Ltd., Beijing, China). The absorbance values for SOD, POD, and CAT were 560 nm, 470 nm, and 405 nm, respectively.

#### 4.2.4. Determination of Soluble Sugar Content

We measured the content of soluble sugar by the anthrone colorimetric method [57]. A total of 0.1 g of plant sample was ground and placed into a 100 mL volumetric flask, then the sample was diluted to volume with distilled water. The solution was heated in a boiling water bath (20 min) and then filtered. 1 mL of the filtrate was collected and mixed with 5 mL of anthrone: 80% H_2_SO_4_ (1:1000, *w*/*v*). The absorbance was measured at 620 nm.

#### 4.2.5. Determination of Soluble Protein Content

We used the Bradford method [58] for soluble protein quantitation. The protein reagent was prepared as follows: 100 mg of Coomassie Blue G250 was dissolved in 50 mL of ethanol (95%), and 100 mL of phosphoric acid (85%, *w*/*v*) was added to the solution. The resulting solution was finally diluted with distilled water to a volume of 1000 mL.

0.1 g of plant sample was homogenized with distilled water on an ice bath and then centrifuged (3000 rpm, 10 min, room temperature), following which 0.1 mL of the supernatant was collected and mixed well with the protein reagent via gentle vortex mixing. Two minutes later, the absorbance was measured at 595 nm.

### 4.3. Transcriptomics Analysis

We used the Plant RNA Purification Reagent (Thermo Scientific, Shanghai, China) to extracted total RNA from plant samples in the control, AN1, and AN40 groups according to the manufacturer’s instructions. The genomic DNA was removed using DNase I (Takara Bio, Beijing, China). The purity and integrity of RNA samples were determined using a NanoDrop 2000 spectrophotometer (Thermo Scientific, Shanghai, China) and Agilent 2100 bioanalyzer (Agilent, Beijing, China), respectively. Qualified RNA samples (1.8≤OD260/OD280≤2.2, OD260/OD230≥2.0, RIN≥6.5, and 28S:18S≥1.0) were used to construct the sequencing library. After mRNA isolation and fragmentation, double-stranded cDNA was synthesized using the SuperScript double-stranded cDNA synthesis kit (Thermo Scientific, Shanghai, China) and random hexamer primers (Illumina, Shanghai, China). End repair and A base addition were then performed on the synthesized cDNA according to the library construction protocol of Illumina. Fragments approximately 300 bp in length were collected using Agencourt AMPure XP Reagent (Beckman, Shanghai, China) and then subject to 15 cycles of PCR amplification. Sequencing was performed using Illumina HiSeq Xten (Illumina, Shanghai, China) after quantification. The transcriptional experiments were run in triplicate.

Paired-ends were trimmed and quality controlled using Seqprep (https://github.com/jstjohn/SeqPrep, accessed on 8 January 2025) and Sickcle (https://github.com/najoshi/sickle, accessed on 8 January 2025) with default parameters, respectively. The clean reads were then mapped to the reference genome using HiSat2 (https://daehwankimlab.github.io/hisat2/, accessed on 8 January 2025). Gene abundance was quantified using RSEM (http://deweylab.github.io/RSEM/, accessed on 8 January 2025) and Fragments Per Kilobase of transcript per Million mapped reads (FPKM) was used to measure the gene expression level. Differential expression analysis was performed using DESeq2 (http://bioconductor.org/packages/stats/bioc/DESeq2/, accessed on 8 January 2025) and genes with $\left|\log_{2}FC\right|>1$ and FDR(FalseDiscoveryRate)<0.05 were considered as DEGs. Go-based and KEGG-based enrichment analysis were performed using Goatools (https://github.com/tanghaibao/GOatools, accessed on 8 January 2025) and KOBAS (http://kobas.cbi.pku.edu.cn/home.do, accessed on 12 March 2024), respectively.

### 4.4. Metabolomics Profiling

Samples measuring 50 mg were collected from the control (six replicates), AN1 and AN40 groups and the metabolites were extracted using methanol–water solution (4:1, *v*/*v*) with 0.02 mg/mL L-2-chlorophenylalanine as an internal standard. The mixture was ground by a frozen tissue grinder (Wonbio-96c, Shanghai Wanbo biotechnology Co., Ltd., Shanghai, China) for 6 min (−10 °C, 50 Hz), and then an ultrasonic extraction (30 min, 5 °C, 40 kHz) was performed. The obtained sample was kept at −20 °C for 30 min to precipitate proteins and then centrifuged (13,000× *g*, 15 min, 4 °C). The supernatant was then collected and fed to an UHPLC-Q Exactive HF-X system (Thermo Fisher Scientific, Shanghai, China) for UHPLC-MS/MS (Ultra High Performance Liquid Chromatography-Tandem Mass Spectrometry) analysis, and the chromatographic and mass spectrometry conditions were set according to reference [59]. The metabolome analysis had six replicates.

The raw LC-MS (Liquid Chromatography-Mass Spectrometer) data were pre-processed by Progenesis QI (Waters, Shanghai, China) and the HMDB (https://www.hmdb.ca/, accessed on 8 January 2025) and Metlin (https://metlin.scripps.edu/, accessed on 8 January 2025) databases as well as a self-built database were used for metabolites identification and analysis. DAMs were identified according to the fold change (FC) value, VIP, and *p*-value of *t*-test. Metabolites whose VIP ≥ 1 and *p*-value < 0.05 were considered as DAMs. The DAMs were then annotated into metabolic pathways through the KEGG database [60], and the python package scipy.stats [61] was used to perform the pathway enrichment analysis.

### 4.5. qRT-PCR Analysis

The reaction mix (20 µL) contained 10 µL of SYBR^®^qPCR Mix (Toyobo, Shanghai, China), 2 µL cDNA template, 0.8 µM forward primer, 0.8 µM reverse primer, 0.4 µL 50 × ROX refernce dye, and 6 µL ddH_2_O. The amplification was conducted using a LightCycler^®^96 system for 35 cycles of 15 s at 95 °C, 30 s at 55 °C and 30 s at 72 °C. The relative expression of per DEGs was evaluated by the $2^{-\Delta \Delta C_{T}}$ method [62].

### 4.6. Statistical Analysis and Visualization

We used ANOVA (Analysis of Variance) to evaluate the statistical significance of the physiological differences between the control and treatment groups and the difference was considered as significant if p<0.05. The analysis was conducted using SPSS version 27. The visualization of the analysis results of transcriptomic and metabolomic data was performed on the Majorbio (cloud.majorbio.com, accessed on 8 January 2025) online platform.

## 5. Conclusions

This study combined a physiological approach with multi-omics analysis to investigate the stress response and tolerance mechanisms of rice under aniline stress. Our research results indicated that the toxicity of high concentrations of aniline significantly inhibited rice growth and altered the levels of antioxidant enzymes, MDA, H_2_O_2_, and soluble substances. Metabolomics and transcriptomics analyses revealed changes in metabolites and genes in rice under aniline stress. The DEGs and DAMs were primarily associated with functions such as lignin synthesis, antioxidant activity, signal transduction, membrane remodeling, and nitrogen supply. The phenylpropanoid biosynthesis, amino acid, lipid, and purine metabolism pathways were key metabolic pathways involved in rice’s response to aniline stress. Considering the limited ability of plants to absorb and transform aniline, we believe that the damage caused by aniline to plants was primarily oxidative damage.

It is noteworthy that in a 1 mg/L solution of aniline, the growth of rice seedlings was not inhibited but rather promoted. Considering the growth-promoting effect of IAA on plants, we believe that the accumulation of IAA was the primary reason why the rice in the AN1 group developed better than that in the AN0 group.

Our research contributes to a more comprehensive understanding of the physiological and biochemical changes and stress resistance mechanisms of rice under aniline stress, while also providing insights for stress resistance studies in other crops. Future research can build upon this work to further explore the long-term effects of aniline exposure on rice, aiming to gain a more comprehensive understanding of the adaptive mechanisms of rice under aniline stress and potential genetic or phenotypic changes. Additionally, comparing the stress response mechanisms identified in this study with other abiotic stress conditions (such as drought or salinity) will be an intriguing and meaningful research direction. By investigating the cross-tolerance potential of rice plants, we can provide new insights and targets for crop improvement.

## Data Availability

Data is contained within the article and Supplementary Materials.

## Supplementary Materials

The following supporting information can be downloaded at: www.mdpi.com/article/10.3390/ijms26020582/s1.

## Abbreviations

The following abbreviations are used in this manuscript:

AN	aniline
MDA	malondialdehyde
H_2_O_2_	hydrogen peroxide
SOD	superoxide dismutase
POD	peroxidase
CAT	catalase
AN1	AN1 represents an aniline solution with a concentration of 1 mg/L, and so on.
AN0	AN0 represents the control group, where 0 indicates zero aniline content.
SP	Soluble Protein
QC	Quality Controlled
GC	Guanine and Cytosine
DEGs	Differentially Expressed Genes
GO	Gene Ontology
KEGG	Kyoto Encyclopedia of Genes and Genomes
qRT-PCR	Quantitative Real-time Polymerase Chain Reaction
OPLS-DA	Orthogonal Partial Least Squares-Discriminant Analysis
DAMs	Differentially Accumulated Metabolites
VIP	Variable Importance in the Projection
C4H	cinnamate 4-hydroxylase
CAD	cinnamylalcohol dehydrogenase
HCT	Hydroxycinnamoyl-CoA shikimate/quinate hydroxycinnamoyltransferase
CCR	Cinnamoyl-CoA reductase
ROS	reactive oxygen species
CoA	coenzyme A
PRX	peroxidase
TCA	tricarboxylic acid
GST	glutathione S-transferase
GSH	glutathione
IAA	indole-3-acetic acid
ALDH	acetaldehyde dehydrogenase
PA	phosphatidic acid
PG	phosphatidylglycerol
PC	phosphatidylcholine
AC	adenylate cyclase
GC	guanylate cyclase
cGMP	cyclic guanosine monophosphate
cAMP	cyclic adenosine monophosphate
FPKM	Fragments Per Kilobase of transcript per Million mapped reads
FDR	False Discovery Rate
UHPLC-MS/MS	Ultra High Performance Liquid Chromatography-Tandem Mass Spectrometry
FC	Fold Change
LC-MS	Liquid Chromatography-Mass Spectrometer
ANOVA	Analysis of Variance
